# Treatment of Spinal War Traumatisms

**Published:** 1919

**Authors:** 


**Treatment of Spinal War Traumatisms.** By CH. VILLANDRE. *La Presse Médicale*, November 7, 1918.

The author speaks of the importance of rational treatment in cases of wounds of the spine, and gives a detailed account of such wounds.

1. *Nervous Shock.* A more or less pronounced state of nervous shock usually follows spinal traumatism, demanding immediate and methodical treatment. In cases where such condition persists in spite of the treatment, the accompanying factors, hemorrhage, visceral complications, etc., should be diagnosed, and surgical intervention resorted to as soon as possible.

2. *Visceral, Paraspinal and Vascular Lesions.* Patients suffering from joint medullar, pleural and pulmonary lesions should not be kept at C. C. S'. s., but submitted to surgical treatment with the least possible delay.

3. *Infection.* A spinal wound may become infected either by shell splinters, fragment of earth and clothing, or by pathogenic agents from the skin. Purulent meningitis is the most dangerous complication occurring in wounds of the spine. Rapid evacuation of the cases concerned, excision of the wound and removal of foreign bodies, sequestra, etc., are imperative. When primitive suture is impossible, owing to the age or extent of the wound, Carrel's method of disinfection should be applied.

4. *Other Complications.* *a*) Eschars : All sources of local infection should be carefully removed and the patient's position changed every hour. Eschars may be either dressed 3 to 4 times a day with aseptic gauze, exposed to sun or hot air, or treated by means of the solution recommended by Roussy :

| | |
|---|---|
| Gomenol. . . . . . . . . . . . . . . . . . . . . . . | 200 |
| Eucalyptol. . . . . . . . . . . . . . . . . . . . . | 100 |
| Tinc. Iodine. . . . . . . . . . . . . . . . . . . . | 20 |
| Ether. . . . . . . . . . . . . . . . . . . . . . . | 1000 |

*b*) Urinary infection may be avoided or at least localized by regular, strictly aseptic soundings, and vesical, antiseptic irrigation.

*c*) Cases with pleuro-pulmonary complications should be immediately placed in warm, well-aired wards, and carefully examined twice a day. Patients should be removed to special hospitals as soon as their condition permits.

*Surgery.* The treatment of spinal wounds requires :

1. Large, superheated wards with special equipment ; (*a*) wide, comfortable beds ; (*b*) water-beds ; (*c*) total suspension devices.
2. Special operative tables, radioscopic installation, etc.
3. An especially trained personnel.

For these reasons it could not be undertaken in field hospitals and C.C.S.'s.

The author further discusses the value of early surgical action and quotes the opinion of various observers, such as Gosset, Derache, etc. Ch. Villandre himself believes that pathologic anatomy is of greater assistance in judging as to the advisability of early operation than clinical data. In cases of spinal traumatism, pathologic anatomy shows that any lesion resulting in the compression of the spinal cord demands early operation. On the other hand, from a bacteriological standpoint, all foreign bodies sequestra, etc., must be removed within the first 6 to 8 hours and suture performed, in order to avoid primary or secondary infection.

As reported by Roussy, Lhermitte, and Sharpe, medullary suture improves the general condition of the patient as regards both trophic and sensitive troubles. Lortat-Jacob, Girou and Ferrand record partial recovery of the spinal motility and sensibility after suture. These observations point out the advisability of suture of the spinal cord whenever it is possible ; unfortunately, however, in a large proportion of wounds of the spine met with in this war, the damage done by the projectile is such that the surgeon must confine himself to mechanical disinfection and removal of the causes of medullary compression. Local anesthesia is recommended.

*Evacuation.* The patients should be evacuated as early as possible to the nearest neurological center.

